# Microbially Induced Calcite Precipitation Employing Environmental Isolates

**DOI:** 10.3390/ma9060468

**Published:** 2016-06-15

**Authors:** Gunjo Kim, Heejung Youn

**Affiliations:** Department of Civil Engineering, Hongik University, 94, Wausan-ro, Mapo-gu, Seoul 04066, Korea; rmsdbrto@naver.com

**Keywords:** MICP, microbe, calcite precipitation, urease activity, environmental isolates

## Abstract

In this study, five microbes were employed to precipitate calcite in cohesionless soils. Four microbes were selected from calcite-precipitating microbes isolated from calcareous sand and limestone cave soils, with *Sporosarcina pasteurii* ATCC 11859 (standard strain) used as a control. Urease activities of the four microbes were higher than that of *S. pasteurii*. The microbes and urea–CaCl_2_ medium were injected at least four times into cohesionless soils of two different relative densities (60% and 80%), and the amount of calcite precipitation was measured. It was found that the relative density of cohesionless soils significantly affects the amount of calcite precipitation and that there is a weak correlation between urease activity and calcite precipitation.

## 1. Introduction

Increasing attention is being paid to eco-friendly soil improvement technologies that do not harm the environment, unlike conventional soil improvement methods. The idea of using microbes to apply to biogeotechnical engineering was first introduced in 1992 [1]. Later, further research was carried out to extend the possibility of employing microbes in the geotechnical field as one of such eco-friendly technologies [2,3,4,5,6]. The idea behind the method is that microbes catalyze chemical reactions and produce sediments that can coat or/and bind grains. The induced calcite precipitation was called microbially induced calcite precipitation (MICP). MICP applications can be diverse and include soil strength improvement, permeability reduction, seismic remediation, and surface erosion control.

Soil strength improvement by biogrouting has attracted the attention of geotechnical engineers and researchers for many years. Isotropically consolidated undrained compression (CIUC) triaxial tests were performed with MICP-treated Ottawa sand, and soil behavior was found to be similar to gypsum-cemented specimens [3]. Direct shear tests and California Bearing Ratio (CBR) tests of treated sand were conducted with growing, resting, and dead *Sporosarcina pasteurii* [7]. They found that geomechanical properties were effectively improved by an application of growing *S. pasteurii*. However, measured increases in friction angle and bearing strength were small for dead and resting *S. pasteurii*. The possibility of soil improvement of unsaturated sand column was introduced using surface percolation of MICP [8], as the strength of the sand column increased to 390 kPa without crust formation on the top of the column. Later, they performed large scale experiments and found that the surface percolation method was more suitable for coarse sand of high permeability [9]. Electro-biogrouting was adopted, involving electro-kinetic injection of bacteria, urea, and calcium ions for the improvement of soft clay [10]. Undrained shear strength increased up to 1080% (52–65 kPa) after seven days. The performance of two microbes (*S. pasteurii* and *Idiomarina insulisalsae*) was compared by a uniaxial compression test and a splitting tensile strength test for different curing times. The results revealed that *I. insulisalsae* was more efficient than *S. pasteurii* and induced the compressive strength by up to 158% [11]. The bacteria-catalyzed MICP process enhanced the unconfined compressive strength (UCS) up to 2.04 MPa, about five times the strength from the process catalyzed by urease (0.43 MPa) [12]. The effect of a suspension of intact *Bacillus* sp. cells, suspension of washed bacterial cells, and culture liquid without bacterial cells on calcite precipitation was investigated [5]. The results showed that the adsorption of urease activity of sand treated with a suspension of washed cells was 5–8 times greater than that treated with urea–CaCl_2_ liquid, and that the unconfined compressive strength of the sand treated with a suspension of washed cells was 1.7 times that of sand treated with urea–CaCl_2_ liquid.

The permeability of soil can be reduced by filling the void with calcite precipitate [6,13,14]. The permeability of sand was reduced from 1.0 × 10^−4^ m/s to 1.6 × 10^−7^ m/s by forming 0.6 g/cm^2^ crust on the sand surface [13]. The measured modulus of rupture (maximum bending stress) of the crust was 35.9 MPa, which was comparable with that of limestone. A calcium salt solution was introduced above and below sand surface, and the location of calcite formation was compared [6]. When the solution was applied below the sand surface, MICP took place inside the sand; on the other hand, when the solution was applied above the sand surface, a thin crust formed on the surface. In both cases, the permeability of the sequentially treated sand was identically reduced to 14 mm/day, and the quantities of precipitated calcium were 0.15 g/cm^2^ and 0.6 g/cm^2^.

Field applications should build on an integrated knowledge of microbiology, ecology, and geochemistry, as well as geotechnical engineering. The feasibility of bio-grouting of sand was confirmed through laboratory tests, followed by large-scale tests in 100 m^3^ volumes [15], and later the feasibility of denitrification during calcite precipitation-inducing urea hydrolysis was confirmed [16]. However, the rate of calcite precipitation by denitrification was lower than that by the urease process. A field and modeling study to seal the fractured rock were conducted using *S. pasteurii*, and a significant reduction in permeability was achieved over 17 h of treatment [17]. Furthermore, full-scale grouting of a gravel layer borehole was carried out using a number of technologies developed through laboratory testing [18,19,20].

Previous studies involved injection of exogenous bacteria into the soil for calcite precipitation, but naturally found that bacteria can also precipitate significant amounts of calcite [21,22,23,24]. Indigenous microorganisms were used to precipitate calcite in liquefiable saturated soils both, in laboratory and *in situ*. In the cone penetration test, the measured resistance was about 6 MPa [22].

However, the isolation of ureolytic soil bacteria and the testing of their activity have not been reported as much. In this study, the amount of calcite precipitation was measured for the MICP-treated sand, with environmental isolates from calcareous sand and limestone cave soils. Isolates with urease activity higher than that of *S. pasteurii* ATCC 11859 were selected. The MICP-treated sand was analyzed with a Scanning Electron Microscope (SEM) and Energy Dispersive Spectroscopy (EDS) to verify calcite precipitation.

## 2. Materials and Methods

### 2.1. Isolation and Maintenance of Microbial Strains

Water and soil samples were taken from calcareous sand (Daechon beach, Daechon, Korea) and limestone cave soils (Hwanseongul, Samcheok, Korea) to isolate calcium carbonate-producing microbes. Urease activity was used for microbial selection. *S. pasteurii* ATCC 11859, a standard strain in this study, was purchased from Korean Collection for Type Cultures (KCTC, Daejeon, Korea). *S. pasteurii* stocks were maintained at −80 °C in 20% glycerol, and the cells were activated by cultivation in tryptic soy broth (TSB; Difco Laboratories, Detroit, MI, USA, pH 7.3) containing urea (2%, w/v), for 2–3 days at 28 °C before use. Bacterial strains were isolated from two natural environments: 35 strains from calcareous sand and 13 strains from limestone cave soils. All samples (1 g) were transferred to 9 mL of sterile 0.85% NaCl (Samchun chemical, Gyeonggi-do, Korea) and mixed for 60 s. The samples were then serially diluted (10-fold) with 9 mL of sterile 0.85% NaCl, and 0.1-mL samples or diluents were plated on urea–CaCl_2_ medium. Urea–CaCl_2_ medium containing 3 g/L nutrient broth (Difco), 10 g/L NH_4_Cl, and 2.12 g/L NaHCO_3_ (equivalent to 25.2 mM) was prepared and adjusted to pH 6.0 with 6 N HCl (Duksan Pure Chemical Co., Ansan-si, Korea). Filter-sterilized 3.7 g/L CaCl_2_ × 2H_2_O (Daejung chemical, Goryeong-gun, Korea) and 20 g/L urea (Junsei Chemical, Tokyo, Japan) were added to autoclaved urea–CaCl_2_ medium after its cooling to 50 °C [25]. The plates were incubated at 28 °C for 5–7 days. Individual colonies with an obvious crystal formation were selected and purified via streaking on urea–CaCl_2_ medium. The colonies were cultivated in urea-containing (2%, w/v) TSB, at 28 °C for 2–3 days, and urease activities were measured, as described below.

### 2.2. Measurement of Urease Activity

Urease activity was determined by measuring the amount of ammonia released from urea in phenol-hypochlorite urease assay [26]. Bacterial cultures were centrifuged at 6000× *g* for 10 min at 4 °C. Supernatants were discarded, and cell pellets were washed twice with 50 mM 4-(2-hydroxyethyl) piperazine-1-ethanesulfonic acid (HEPES, pH 7.5; H3375-25 g, Sigma-Aldrich, St. Louis, MO, USA). Bacteria were sonicated (three pulses of 30 s each) on ice and centrifuged at 6000× *g* for 10 min at 4 °C. Supernatants (0.5 mL) were added to urease buffer (50 mM HEPES, 25 mM urea), 1 mL of final volume, and incubated at 37 °C for 20 min. The reaction was stopped by transferring an aliquot to 15-mL conical tubes (SPL, Gyeonggi-do, Korea) containing 1.5 mL of solution A (containing, per liter, 10 g phenol and 50 mg sodium nitroprusside). An equal volume (1.5 mL) of solution B (5 mg/mL NaOH, 0.044% *v*/*v* NaClO) was added, and the contents were mixed. Following incubation at 37 °C for 30 min, absorbance was measured with a microplate reader (Multiskan EX, Vantaa, Finland) at a wavelength of 620 nm. A standard curve of optical density (OD) was determined with ammonium chloride solutions at 0.0001%, 0.001%, 0.01%, 0.1%, 0.5%, and 1% (w/v) concentrations. Urease activity can be indirectly assessed with the ammonium chloride concentration calculated from OD at 620 nm. The correlation between OD620 nm and ammonium chloride concentration is shown in the following equation.

(1) $$OD_{620nm}\;=\;0.219\;\times ammonium\;chloride\;concentration\;(\%)\;+\;0.0557$$

### 2.3. Bacterial Identification by 16S rRNA Sequencing

Bacterial strains with urease activities exceeding that of the standard strain *S. pasteurii* were selected and identified by 16S rRNA sequencing. 16S rRNA sequencing was performed commercially (Solgent Co. Ltd., Daejeon, Korea). Briefly, DNA isolation was performed using purification beads (Solgent). Universal primers 27F (5′-AGA GTT TGA TCC TGG CTC AG-3′) and 1492R (5′-GGT TAC CTT GTT ACG ACT T-3′) were used to amplify community 16S rRNA gene. Each PCR mixture contained Solg™ EF-Taq DNA polymerase (Solgent) and was prepared according to the manufacturer’s instructions. PCR amplification was performed using GeneAmp^®^ PCR system 9700 (Applied Biosystem, Foster City, CA, USA). PCR conditions were as follows: 95 °C for 2 min (first cycle), followed by 95 °C denaturation for 20 s, 56 °C annealing for 40 s, and 72 °C annealing for 90 s for a total of 30 cycles, followed by a 3-min extension at 72 °C. PCR products were purified with Solg™ PCR purification kit (Solgent) according to the manufacturer’s instructions. The purified bacterial amplicons were cloned using T-Blunt™ PCR Cloning Kit (Solgent). Cloned sequences were determined with ABI 3730XL DNA Sequencer (Applied Biosystem). Sequence-based close relatives and microbial phylogenetic affiliations were verified using a BLAST search (best hit similarity ≥98%) at the National Center for Biotechnology Information [27].

### 2.4. Bacterial Inoculation and MICP Treatment

Isolated bacteria were inoculated into urea–CaCl_2_ medium to 10^8^–10^9^ CFU/mL final concentration and maintained at −80 °C in 20% glycerol. For propagation, stock solutions were activated at room temperature for 10 min before use. Next, the bacterial strains were inoculated into 35 mL of urea–CaCl_2_ medium and cultivated at 28 °C for 2–3 days before inoculation into sand specimens. Activated cultures (10^8^–10^9^ CFU/mL) in 35 mL of urea–CaCl_2_ medium were used to inoculate sand for MICP treatment with 500 mL of urea–CaCl_2_ medium.

### 2.5. Calcite Precipitation

**Figure 1:**
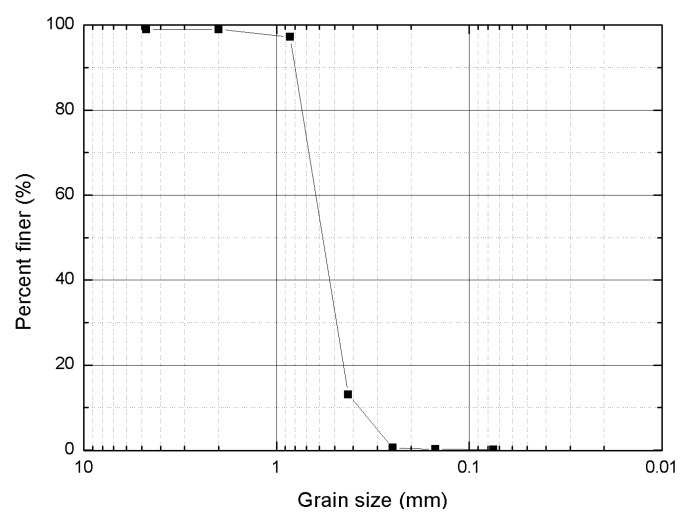
Grain size distribution of Jumunjin sand.

Figure 1 shows a Jumunjin sand grain size distribution curve, which indicates that the sand was classified as SP (poorly graded sand) according to the Unified Soil Classification System (USCS) [28]. The measured specific gravity of the sand was 2.64, and the maximum and minimum dry unit weights were 16.0 kN/m^3^ and 13.5 kN/m^3^, respectively.

**Figure 2:**
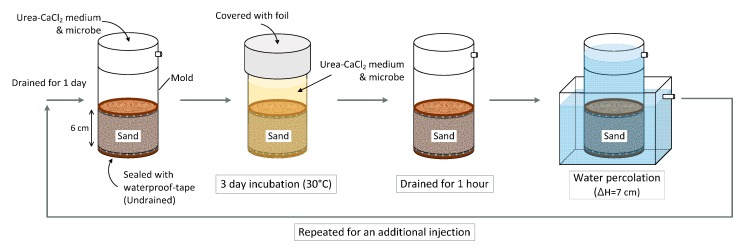
Calcite precipitation process.

The amount of calcite precipitation in Jumunjin sand was measured under different conditions that involved different relative densities, the number of injections, and different microbial strains used. The MICP process is shown in Figure 2. A relative density of Jumunjin sand set to 60% or 80% in a specimen preparation mold. The waterproof-tape was used to seal the bottom of the mold, and a urea–CaCl_2_ medium (500 mL) and microbe solution (5 mL) were added to the top of the sand for MICP treatment. MICP-treated sand was incubated for 3 days at 30 °C, with mold top covered with foil. The sand specimen was then drained for 1 h at room temperature. Following this, water percolated the sand with constant difference in hydraulic head of 7 cm. The MICP-treated sand was then drained for a day and ready for the second cycle of MICP treatment. In the consecutive cycles, a new urea–CaCl_2_ medium and microbe solution was injected, and calcite precipitation of MICP-treated sand after second injections was measured. The aforementioned procedure was repeated with five microbial strains and two relative densities of sand specimen.

### 2.6. Calcium Carbonate Measurements

**Figure 3:**
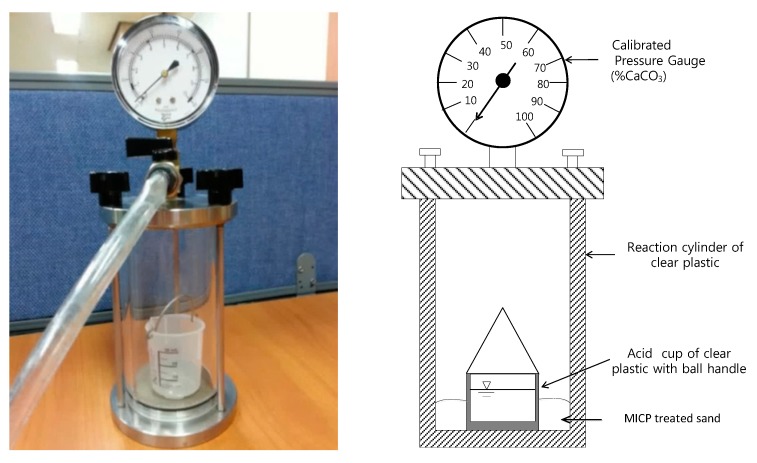
Calcium carbonate content chamber.

**Figure 4:**
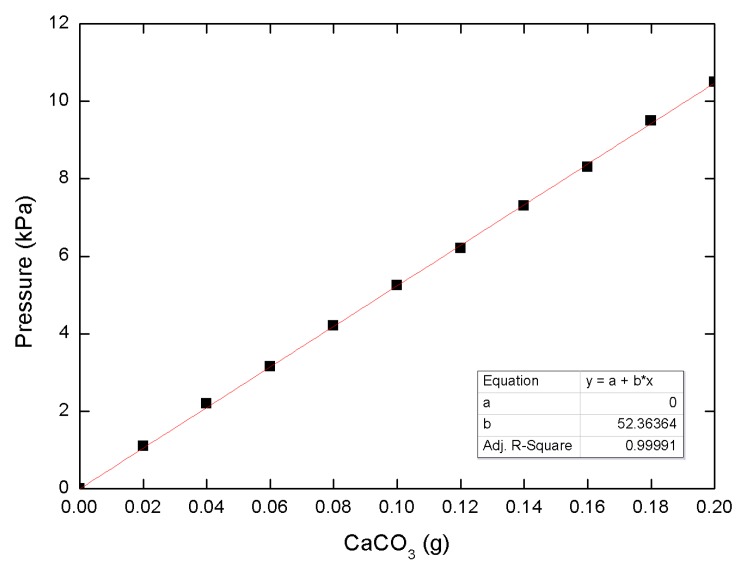
Gas pressure–CaCO_3_ relationship.

The amount of calcium carbonate precipitation was measured using ASTM D4373 [29]. This standard method utilizes a reaction cylinder, a cup filled with hydrochloric acid (HCl), and pressure gauge (see Figure 3). Briefly, the sand sample was poured into the reaction cylinder and a cup filled with HCl was placed in the cylinder. The reaction cylinder was closed tight to prevent gas leakage, and was tilted so that the acid reacted with the treated sand. Carbon dioxide (CO_2_) was produced in the cylinder in the course of a chemical reaction between the calcium carbonate sand deposit and HCl. Gas (CO_2_) pressure was related to the amount of calcium carbonate using a calibration curve. The calibration curve was created using 20 mL 1 N HCl (80 mL HCl in 720 mL distilled water) and different amounts of calcium carbonate (0.2 g, 0.4 g, 0.6 g, 0.8 g, and 1.0 g) (CaCO_3_: Showa Chemicals Inc., Tokyo, Japan). The resulting curve is presented in Figure 4. Further details of the standard method can be found in ASTM D4373 [29].

The MICP-treated sand was dried for two days before precipitated calcium carbonate measurements. The dried sand (10 g) and 1 N HCl (20 mL) were allowed to react for approximately 10 min, since no reaction occurred after that time period. The amount of calcium carbonate was estimated using the calibration curve presented in Figure 4. This process was repeated ten times, for CaCO_3_ precipitation measurements from 100-g specimens. Precipitated carbon carbonate was estimated for the same volume of sand samples (148.54 g/100 cm^3^ of 60% relative density sand, and 154.49 g/100 cm^3^ of 80% relative density sand).

### 2.7. Scanning Electron Microscopy (SEM) & Energy Dispersive Spectroscopy (EDS)

SEM allows an examination of sand surface particles after conversion of secondary electrons generated by an interaction of an electron beam and specimen into a video signal. SEM can be used to analyze the texture of an object surface and particle shapes. SEM was used to verify calcium carbonate precipitation in MICP-treated sand dried for two days. Carbon tape was glued to a specimen holder to immobilize sand grains and allow the analysis. Some of the dried sand was transferred to the holder and the sample was coated by platinum sputtering using a microscopy coater. Calcium carbonate was observed by changing the magnification of the electron microscope.

Quantitative analysis of elemental composition of the microbially treated sand was performed with EDS. Crystals precipitated on the surface of silica sand grains were observed in the SEM and were found to be mostly SiO_2_ and CaCO_3_ by EDS.

## 3. Results and Discussion

### 3.1. Bacterial Isolation Based on Urease Activity Determinations

**Figure 5:**
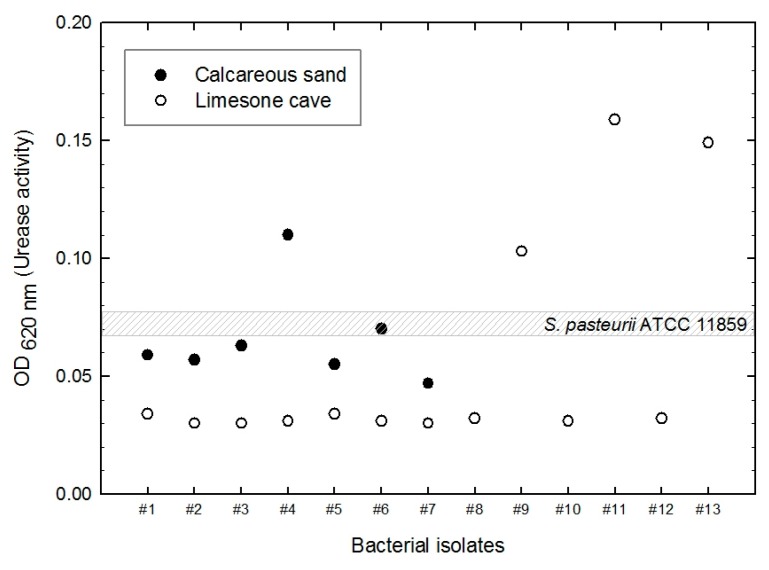
Urease activity of 20 microbial isolates.

Urease activity was tested in 20 environmental isolates (7 strains from the calcareous sand and 13 strains from the limestone cave soils) that displayed obvious crystal formation; the results are shown in Figure 5. Urease activities of the isolates and *S. pasteurii* ATCC 11859 standard strain were measured by the phenol-hypochlorite urease assay. Four isolates showed higher urease activity than the standard strain, namely, sample number 4 isolated from calcareous sand and samples 9, 11, and 13 isolated from the limestone cave. Their OD_620_ values were 0.110, 0.103, 0.159, and 0.149, respectively.

Four isolates with high level of urease activity were selected for further analysis and were identified by 16S rRNA sequencing (Table 1). Sample 4 isolated from calcareous sand was confirmed as *Staphylococcus saprophyticus* subsp. *saprophyticus* (99% identity). Samples 9, 11, and 13 from limestone cave were identified as *Sporosarcina globispora*, *Bacillus lentus* strain NCIMB8773, and *Sporosarcina* sp., respectively (identity >98% for all cases).

### 3.2. Calcium Carbonate Measurements

**Figure 6:**
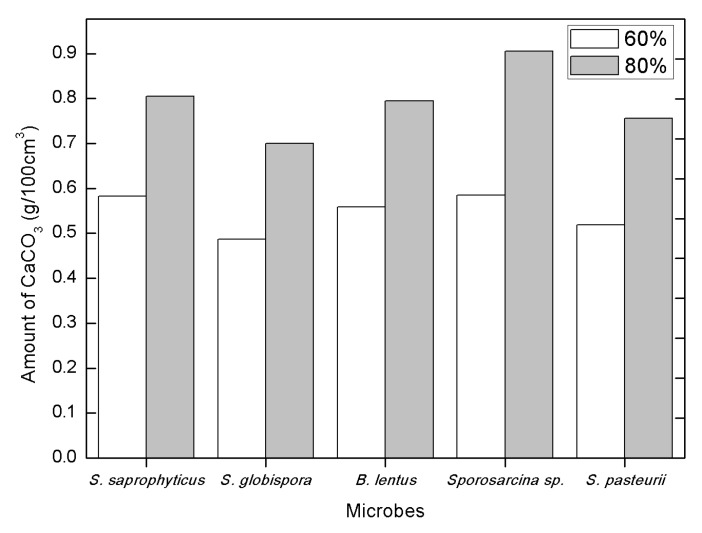
Amount of calcium carbonate.

The amount of calcium carbonate precipitation in MICP-treated sand was measured for two relative soil densities, and the results are presented in Figure 6. It should be pointed out that the amount of calcium carbonate in the sand was measured and calculated for the same volume (100 cm^3^), not for the same weight. Calcium carbonate precipitation in 80% sand was approximately 1.4 times that of 60% sand for all microbes tested.

Results summarized in Table 2 show the amount of calcite precipitation, and their relative rankings. Based on urease activity measurements, *B. lentus* was expected to rank highest in calcite precipitation, but it ranked third in calcite amount. On the other hand, *Sporosarcina* sp. and *S. globispora*, which ranked second and fourth with respect to the measured urease activity, respectively, ranked highest and lowest, respectively, in calcite precipitation. It can be concluded that the urease activity does not correlate well with calcite precipitation. It is also interesting to note that calcite precipitation was measured more in the higher density sand, indicating that MICP treatment is effective in small void soils (small particle size). It seems that calcite precipitation on sand grains are likely to be washed away during water percolation, and the pore water velocity through voids affect the total amount of calcite precipitation. With a higher relative density, there is a greater chance that calcite precipitation stays in the voids or sticks to the sand grain without washing away. There is no difference in rank for different relative density.

### 3.3. Scanning Electron Microscopy (SEM) & Energy Dispersive Spectroscopy (EDS)

**Figure 7:**
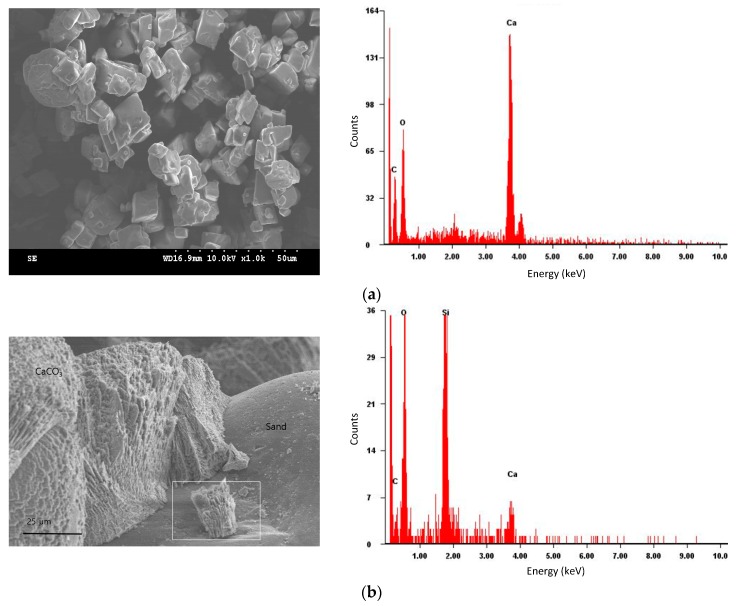
SEM and EDS analyses of (**a**) calcium carbonate powder; and (**b**) crystalline substances on silicate sand grains.

Scanning electron microscopy analysis was carried out on MICP-treated sand specimens, but no differences were observed when different microbes and relative soil densities were examined. Hence, SEM and EDS results provided here are representative of all of our sand specimens and calcium carbonate powder (Figure 7). Interestingly, unlike with calcium carbonate powder, the carbonate precipitate on sand grain surface was not cubical but, rather, crystalline. EDS determined the crystalline elements to be CaCO_3_.

## 4. Conclusions

Calcite precipitation of four environmental isolates and *S. pasteurii* ATCC 11859 (standard strain) were measured and compared. Four isolates were selected based on their urease activities, which were higher than that of *S. pasteurii*. Jumunjin sand was microbially treated for two different relative soil densities (60% and 80%), and the amount of calcium carbonate precipitation was measured. SEM and EDS analyses were carried out to verify calcium carbonate precipitation. The following conclusions were drawn:

1. Four isolates (*S. saprophyticus*, *S. globispora*, *B. lentus*, and *Sporosarcina* sp.) were selected from 20 environmental microbes that precipitated calcium carbonate and displayed higher urease activity than *S. pasteurii*.
2. Relative density of cohesionless soils significantly affected the amount of calcite precipitation, which was measured to be approximately 40%–50% higher in 80% relative density soil than that in 60% relative density soil.
3. The amount of calcium carbonate precipitation was greatest for *Sporosarcina* sp., followed by *S. saprophyticus*, *B. lentus*, *S. pasteurii*, and *S. globispora*. The difference between the greatest and smallest was about 17% and 23% for 60% and 80% relative density soil, respectively.
4. The urease activity was highest in *B. lentus*, followed by *Sporosarcina* sp., *S. saprophyticus*, *S. globispora*, and *S. pasteurii*. It appears that the urease activity weakly correlates with calcium carbonate precipitation.
5. SEM analysis identified a crystalline substance on the sand grain surface after MICP treatment. The substance was verified as the calcium carbonate with EDS.

## Figures and Tables

**Table 1:** Identification of microbial strains isolated from limestone cave and calcareous sand.

Sample	No.	Microbes	Identity
Calcareous sand	4	*Staphylococcus saprophyticus* subsp. *saprophyticus*	99%
Limestone cave soils	9	*Sporosarcina globispora*	98%
	11	*Bacillus lentus* strain NCIMB8773	99%
	13	*Sporosarcina* sp.	99%

**Table 2:** Microbe ranking based on the amount of calcite and urease activity.

Microbes	Precipitated Calcite				Rank in Urease Activity
	Relative Density = 60%		Relative Density = 80%		
	Amount of Calcite (g/100 cm^3^)	Rank	Amount of Calcite (g/100 cm^3^)	Rank	
*Sporosarcina* sp.	0.585	1	0.905	1	2
*S. saprophyticus*	0.583	2	0.805	2	3
*B. lentus*	0.559	3	0.794	3	1
*S. pasteurii*	0.519	4	0.756	4	5
*S. globispora*	0.487	5	0.700	5	4
